# Study-Specific Morphological Signs of Non-Carious Tooth Surface Loss in Second Permanent Molars and Caries Experience in a Clinical Sample of Adolescents from Southwest Romania: A Multicenter Cross-Sectional Study

**DOI:** 10.3390/jcm15176931

**Published:** 2026-09-07

**Authors:** Narcis Mihaita Bugala, Denisa Alexandra Al Share, Smaranda-Adelina Bugala, Mihaela Jana Țuculină, Dana Maria Albulescu, Alina Nicoleta Capitanescu, Dragos Cadea, Adrian Macovei, Loredana Selaru, Ancuta Ramona Camen, Ana Maria Rica

**Affiliations:** 1 Doctoral School, University of Medicine and Pharmacy of Craiova, 200349 Craiova, Romania; bugala.mihai@yahoo.com; 2 Faculty of Dental Medicine, Titu Maiorescu University, 031593 Bucharest, Romania; denisaenache88@yahoo.com; 3 Department of Endodontics, Faculty of Dental Medicine, University of Medicine and Pharmacy of Craiova, 200349 Craiova, Romania; r_ana_maria22@yahoo.com; 4 Department of Anatomy, Faculty of Medicine, University of Medicine and Pharmacy of Craiova, 200349 Craiova, Romania; med73danam@yahoo.com (D.M.A.); dragos.cadea@umfcv.ro (D.C.); adrian.macovei@umfcv.ro (A.M.); 5 Department of Otorhinolaryngology, Faculty of Medicine, University of Medicine and Pharmacy of Craiova, 200349 Craiova, Romania; alinacapitanescu@yahoo.com; 6 Department of Paediatrics, Faculty of Medicine, University of Medicine and Pharmacy of Craiova, 200349 Craiova, Romania; loredana.selaru@gmail.com; 7 Department of Occupational Medicine, Faculty of Medicine, University of Medicine and Pharmacy of Craiova, 200349 Craiova, Romania; ancutzaboicea@gmail.com

**Keywords:** adolescents, dental caries, DMF-T, tooth surface loss, tooth wear, second permanent molars, exploratory cross-sectional study, logistic regression

## Abstract

**Background/Objectives:** Morphological signs of non-carious tooth surface loss are clinically heterogeneous, and their prevalence depends strongly on the diagnostic framework and threshold used. This exploratory secondary analysis evaluated a study-specific combined morphological outcome restricted to second permanent molars and its association with cumulative caries experience in a regional clinical sample of adolescents. **Methods:** The analysis included 231 adolescents aged 13–19 years from Dolj, Gorj, and Olt counties, selected from a parent clinical database of 638 participants. The study-specific outcome was coded when a clearly discernible non-carious hard-tissue change compatible with an abrasive, erosive, or abfraction-related morphology was identified on any clinically accessible surface of tooth 17, 27, 37, or 47. This study did not use BEWE, the Smith and Knight Tooth Wear Index, or another validated severity-based tooth wear index; subtype, surface, and severity were not retained separately in the analytical dataset. Dental caries experience was measured using DMF-T. Group comparisons, correlations, and multivariable logistic regression were performed in JASP version 0.96.0. Sensitivity analyses included a participant-level coding audit and a logistic model using DMF-T recalculated after excluding the contribution of teeth 17, 27, 37, and 47. **Results:** The study-specific morphological outcome was present in 50/231 participants (21.6%). A coding audit confirmed that 181 participants had no positive second molar and 50 had exactly one positive second molar; tooth-specific counts were 14, 15, 12, and 9 for teeth 17, 27, 37, and 47, respectively. In the primary model, male sex (adjusted OR = 3.34, 95% CI 1.67–6.71) and DMF-T (adjusted OR = 1.15 per point, 95% CI 1.01–1.31) were associated with the outcome. After excluding the four evaluated second molars from DMF-T, the caries experience association remained positive but borderline (OR = 1.198, 95% CI 1.000–1.435, *p* = 0.049), while male sex remained associated (OR = 3.310, 95% CI 1.647–6.652, *p* < 0.001). **Conclusions:** A modest exploratory association was observed between the study-specific morphological outcome and cumulative caries experience in this clinical sample. The result should not be interpreted as a standardized NCDL prevalence estimate, a causal relationship, or a prediction model. Confirmation using validated whole-mouth tooth wear measures, outcome-specific reliability assessment, and population-based longitudinal data is required.

## 1. Introduction

Dental caries remains a substantial oral-health burden in children and adolescents worldwide [1,2]. A recent systematic review of European adolescents estimated high caries prevalence and emphasized marked age-, tooth-, and surface-level heterogeneity [3]. DMF-T remains widely used to summarize cumulative permanent-dentition caries experience, although it does not measure lesion activity or distinguish when the contributing events occurred [4,5,6,7].

Non-carious tooth surface loss is clinically heterogeneous. Abrasive, erosive, and cervical wedge-shaped changes may have overlapping appearances and multifactorial determinants, and recent evidence continues to show substantial variability in morphological descriptions and diagnostic approaches [8,9,10,11,12,13]. Contemporary adolescent studies commonly use validated frameworks such as the Basic Erosive Wear Examination (BEWE), with explicit severity thresholds and surface/sextant recording [14,15]. The present study did not use such a validated wear index; therefore, its binary endpoint is treated as a study-specific combined morphological outcome rather than as a standardized diagnosis of non-carious tooth wear.

The current secondary analysis was restricted to teeth 17, 27, 37, and 47 because these were the second permanent molars for which the parent database retained tooth-specific morphological records. These teeth were not selected as validated index teeth and are not proposed as a substitute for a whole-mouth or BEWE-based examination. Their use provides a narrow tooth-specific exploratory view only. Because cumulative caries experience also includes these teeth, a sensitivity analysis excluding their contribution from DMF-T was performed to assess possible local measurement overlap. Recent studies of adolescents demonstrate that erosive tooth wear can vary by age, tooth type, sex, caries status, and behavioral exposures, reinforcing the need for age adjustment and cautious interpretation [14,15].

The primary exploratory objective was to determine whether greater cumulative caries experience was associated with higher odds of the study-specific combined morphological outcome after adjustment for age, sex, residential environment, and county. The directional hypothesis for this secondary analysis was that higher DMF-T would be associated with greater odds of a positive morphological outcome. Secondary objectives were to describe the tooth-specific distribution, evaluate the internal coding consistency of the outcome, and assess whether the association persisted when the contribution of teeth 17, 27, 37, and 47 was removed from DMF-T. The analysis was not designed to estimate population prevalence, establish etiology, or evaluate a diagnostic or screening tool.

## 2. Materials and Methods

### 2.1. Study Design, Setting, and Recruitment

This multicenter cross-sectional clinical study was conducted between April 2025 and April 2026 in Southwest Romania. Recruitment occurred in four dental care settings: one school dental office in Gorj County, one school dental office in Olt County, and two centers in Dolj County, comprising one school dental office and the Endodontics Clinic of the Faculty of Dentistry, University of Medicine and Pharmacy of Craiova. These locations served as recruitment and examination settings; irrespective of recruitment site, all standardized study examinations were performed by the same trained examiner described in Section 2.9, rather than by separate site-specific examiners.

Participants were patients who attended the participating dental facilities for clinical examination or treatment during the study period. The same consecutive, non-probability recruitment procedure was intended at all four settings: patients presenting during the recruitment period were screened for eligibility and eligible individuals were invited to participate. No recruitment quotas or predefined numerical targets were established for individual counties or centers. The final analytical database retained county but did not retain recruitment center as a participant-level variable; therefore, the two Dolj centers cannot be separated retrospectively for center-stratified analysis. The source database also did not retain a center-specific count of refusals or center-specific exclusion reasons.

A total of 674 individuals were assessed for eligibility. Thirty-six individuals were excluded because they did not meet one or more predefined eligibility criteria, and 638 participants were included in the parent clinical database. The source dataset retained the aggregate number excluded but not mutually exclusive counts for each exclusion reason; these, therefore, cannot be reconstructed reliably. The present investigation is a post hoc secondary analysis of the parent database and was restricted to the 231 adolescents aged 13–19 years because permanent-dentition DMF-T is more interpretable in this age range and second permanent molar eruption is expected to be more complete than among younger children. The 407 participants aged 6–12 years were not included. The parent database also underlies two companion analyses addressing distinct questions; the present manuscript focuses only on the study-specific combined morphological outcome in second permanent molars.

Because participants were recruited among patients attending dental services rather than through a population-based sampling frame, the analytical sample was considered a clinical sample and was not assumed to be representative of all children and adolescents living in Southwest Romania. This study was reported in accordance with the STROBE recommendations for observational cross-sectional studies [16,17].

### 2.2. Pragmatic Sample Size Estimation and Analytical Sample

The parent study used a pragmatic sample size approach intended to ensure reasonable precision for estimating oral-health prevalence indicators within the clinical sample rather than to provide formal statistical power for a single association model. Using a 95% confidence level, an expected proportion of 50% as the most conservative assumption, and an absolute precision of 4%, the estimated minimum sample size was 600 participants. After allowing for approximately 5% incomplete or unusable clinical records, the recruitment target was set at no fewer than 630 participants; the final parent database comprised 638 participants.

No separate prospective sample size calculation was performed for this secondary adolescent analysis. All 231 eligible adolescents with complete data were included. To limit overfitting in the presence of 50 participants with the study-specific morphological outcome, the primary logistic regression model was deliberately parsimonious and contained six model degrees of freedom.

### 2.3. Eligibility Criteria

Participants in the parent study were eligible if they were aged 6–19 years, attended one of the participating dental facilities during the recruitment period, had at least one CPI-eligible sextant containing a minimum of two fully erupted and clinically assessable permanent teeth, and were able to undergo the standardized oral examination. Only permanent teeth were included in the dental and periodontal study assessments. Written informed consent was provided by a parent or legal guardian for participants younger than 18 years, together with age-appropriate assent where applicable. Participants aged 18 years or older provided their own written informed consent.

Exclusion criteria were fixed orthodontic treatment; severe dento-maxillary anomalies preventing standardized examination; acute oral conditions requiring immediate treatment before completion of this study examination; systemic diseases or medication use known to substantially alter gingival or periodontal findings, including disorders associated with abnormal bleeding, marked immune dysfunction, or gingival enlargement; professional periodontal treatment or systemic antibiotic use within the previous three months; inability to cooperate with the clinical examination; and incomplete clinical records or missing data for the primary study variables.

For the present secondary analysis, participants additionally had to be aged 13–19 years and have complete clinical records permitting evaluation of DMF-T and lesion status for the four second permanent molars (17, 27, 37, and 47). All 231 adolescents meeting these criteria were included.

### 2.4. Clinical Examination Procedures

All examinations were performed under standardized artificial lighting using a plane dental mirror, a blunt-ended probe for visual-tactile caries and hard-tissue assessment, and a WHO periodontal probe (E. HAHNENKRATT GmbH, Königsbach-Stein, Germany) with a 0.5-mm ball tip and standard markings for periodontal screening. Sterile gauze, disposable gloves, infection-control procedures, and standardized clinical recording forms were used throughout. Radiographs were not used for study classification.

Only fully erupted permanent teeth that could be examined reliably were included. Partially erupted teeth and teeth that could not be assessed adequately because of limited clinical access, extensive soft-tissue coverage, or other local conditions were recorded as not assessable and did not contribute to the corresponding index or lesion classification.

### 2.5. Dental Caries Assessment

Dental caries experience was assessed exclusively in the permanent dentition using the Decayed, Missing, and Filled Teeth index for permanent teeth, reported as DMF-T, according to World Health Organization criteria [4]. Primary teeth were not included, and no dmft score was calculated. Caries was diagnosed clinically by visual-tactile examination. Teeth missing for reasons unrelated to caries, including orthodontic extraction, trauma, or congenital absence, were not included in the M component.

DMF-T was treated as cumulative caries experience rather than as a measure of current lesion activity. Caries experience was defined descriptively as DMF-T greater than 0.

### 2.6. Study-Specific Combined Morphological Outcome Assessment

The four second permanent molars (17, 27, 37, and 47) were examined on all clinically accessible occlusal, buccal, lingual/palatal, mesial, and distal surfaces. The operational threshold for a positive finding was a clearly discernible visual-tactile alteration of the external tooth contour beyond normal anatomy that was judged compatible with one of the study-defined non-carious morphological patterns. Abrasive-pattern change was recorded for a smooth or polished defect with flattening or linear surface loss; erosive-pattern change for a smooth rounded loss of contour, shallow concavity, or occlusal cupping; and abfraction-related change for a wedge-shaped or V-shaped cervical defect near the cemento-enamel junction. Findings attributable to dental caries, restorations, developmental defects, fracture, or trauma were excluded. However, no validated minimum depth, severity grade, BEWE score, or Smith and Knight tooth wear index score was recorded. Consequently, this study cannot reliably distinguish mild physiological wear from early pathological wear or provide a standardized tooth wear diagnosis [12,14,18,19,20].

The predominant morphology was identified clinically, but the final analytical database retained only a combined binary tooth-level variable. Subtype, surface-specific location, and severity were not retained separately and cannot be reconstructed retrospectively. For each molar, the variable was coded 1 when at least one qualifying study-specific morphological change was present and 0 otherwise. The participant-level analytical endpoint (database variable NCDL_present) was coded 1 when at least one of the four molars was positive and 0 when all four were negative. Throughout the revised manuscript, this variable is interpreted as a study-specific combined morphological outcome and not as a validated whole-mouth NCDL or tooth wear diagnosis.

### 2.7. Community Periodontal Index Assessment

Gingival and periodontal screening findings were recorded using a full-mouth CPI-based protocol adapted from World Health Organization principles [4,21]. The permanent dentition was divided into six sextants: maxillary right posterior, maxillary anterior, maxillary left posterior, mandibular left posterior, mandibular anterior, and mandibular right posterior. Third molars were excluded.

All fully erupted and clinically assessable permanent teeth were examined at six sites: mesiobuccal, mid-buccal, distobuccal, mesiolingual or mesiopalatal, mid-lingual or mid-palatal, and distolingual or distopalatal. A WHO periodontal probe was used with light probing pressure. A sextant was eligible when it contained at least two fully erupted and clinically assessable permanent teeth; sextants containing fewer than two eligible teeth were excluded from sextant-level scoring.

For each eligible sextant, the highest score identified at any examined site was recorded: CPI 0, no bleeding on probing and no detectable calculus; CPI 1, bleeding on probing without detectable calculus; and CPI 2, supra- or subgingival calculus detected during probing. The participant-level CPI category was defined as the highest score across eligible sextants. No CPI codes 3 or 4 were observed in the study database; consequently, CPI values ranged from 0 to 2.

### 2.8. Gingival Index and Plaque Index Assessment

Gingival inflammation and plaque accumulation were assessed using the Gingival Index described by Löe and Silness and the Plaque Index described by Silness and Löe [22,23]. For each fully erupted and clinically assessable permanent tooth, four surfaces were evaluated: distal, facial or buccal, mesial, and lingual or palatal. Each surface was assigned a score from 0 to 3 according to the original criteria of the respective index.

For each tooth, GI and PI values were calculated as the arithmetic mean of the corresponding surface scores. Participant-level GI and PI values were then calculated as the arithmetic mean of tooth-level scores across all fully erupted and clinically assessable permanent teeth. The values used in the statistical analyses were therefore continuous participant-level mean scores rather than directly assigned global ordinal categories. Surface-level scores were used to calculate tooth-level and participant-level indices but were not retained as separate variables in the final analytical database.

### 2.9. Examiner Calibration and Data Quality

All clinical examinations at all four recruitment settings were performed by the same examiner; therefore, inter-examiner calibration between centers was not applicable. Before participant recruitment, the examiner underwent theoretical and clinical training in the DMF-T, study-specific morphological criteria, CPI, Gingival Index, and Plaque Index. The examiner was not blinded to the other oral findings recorded during the same clinical examination, and this is acknowledged as a potential source of observer-related bias.

Intra-examiner reproducibility was evaluated in a randomly selected subsample of 62 participants re-examined after 7–10 days without access to the initial findings. The available study records preserve only the previously calculated global Cohen’s kappa of 0.86 across categorical clinical recordings; the paired repeated-record dataset and the number of NCDL-positive repeated participants are no longer available. NCDL-specific 2 x 2 agreement, percentage agreement, kappa with 95% CI, and index-specific ICCs therefore cannot be recalculated. The global kappa is reported only as a general historical indicator of examiner consistency and is not used as evidence that the primary study-specific morphological endpoint had established reproducibility. This absence of outcome-specific reliability is treated as a major limitation.

Clinical forms were reviewed for completeness before database entry. The database was subsequently checked for duplicate records, values outside permitted ranges, internal inconsistencies, implausible combinations, and coding errors. Participants were identified using unique numerical study codes, and directly identifying information was not included in the statistical dataset. Restriction of the current analysis to ages 13–19 reduced potential misclassification related to mixed dentition and incomplete second permanent molar eruption. Selection bias remained possible because recruitment used a consecutive clinical sample rather than a probability-based population sample.

### 2.10. Variables and Covariates

The primary outcome was the participant-level study-specific combined morphological outcome (analytical variable NCDL_present). The principal clinical exposure was whole-mouth permanent-dentition DMF-T. Additional oral-health indicators were CPI, GI, and PI. Demographic covariates were age in years, sex, area of residence (rural or urban), and county (Dolj, Gorj, or Olt). Recruitment center was not retained as a participant-level analytical variable. All 231 adolescents had complete data for the variables included in the primary analyses.

### 2.11. Statistical Analysis

Statistical analyses were performed using JASP (Version 0.96.0; JASP Team, University of Amsterdam, Amsterdam, The Netherlands). All tests were two-sided, and *p* < 0.05 was considered statistically significant. Continuous and ordinal variables were summarized using means, standard deviations, medians, interquartile ranges, and minimum–maximum values; categorical variables were summarized using frequencies and percentages. The analysis plan distinguished binary and count outcomes and explicitly evaluated distributional fit, consistent with recommendations for dental caries count data [24,25].

Because the distributions were discrete, bounded, or non-normal, morphological-outcome-positive and morphological-outcome-negative groups were compared using Mann–Whitney U tests. Rank-biserial correlations with 95% confidence intervals were reported as effect sizes. Associations between participant-level study-specific combined morphological outcome and sex, residence, or county were evaluated using Pearson chi-squared tests. Phi or Cramer’s V quantified effect size. Spearman coefficients described monotonic correlations between the study-specific morphological outcome and the oral clinical indicators.

The primary multivariable analysis was binary logistic regression with the study-specific morphological outcome (NCDL_present) as the dependent variable and DMF-T, age, sex, residence, and county as predictors. Adjusted odds ratios, 95% confidence intervals, likelihood-ratio model tests, pseudo-R2 values, variance inflation factors, and AUC were reported. Two additional checks were performed. First, an internal analytical coding-consistency check examined NCDL_count and the four tooth-specific binary variables to verify the unusual observation that each participant with the outcome had exactly one positive second molar. This check was performed within the analytical dataset only and did not compare the derived variables with the original examination forms; it therefore does not constitute a raw-source audit or establish diagnostic validity or outcome-specific reproducibility. Second, to assess possible local overlap between the outcome teeth and cumulative caries experience, DMF-T was recalculated after removing the D/M/F contribution of teeth 17, 27, 37, and 47 (DMFT_excl_2M), and the same adjusted logistic model was repeated with DMFT_excl_2M replacing total DMF-T. These analyses are reported in Supplementary Tables S1 and S2.

A secondary Poisson generalized linear model with a logarithmic link treated DMF-T as a count outcome and included the study-specific morphological outcome and the same demographic covariates [24,25]. Goodness of fit was assessed using deviance and Pearson statistics. Because the Poisson model showed inadequate fit, a Gaussian identity-link model was used as a sensitivity analysis to estimate the adjusted mean DMF-T difference. PI, GI, and CPI were not entered simultaneously into the primary logistic model because of their strong intercorrelations and the limited number of NCDL events. Secondary bivariate analyses involving these indices were considered exploratory. No formal multiplicity adjustment was applied; exact *p*-values and effect sizes were therefore emphasized.

This study uses an age-restricted subset of the same parent clinical database as two companion manuscripts examining CPI–DMF-T associations and the joint multivariable structure of DMF-T, CPI, PI, and GI. The present manuscript addresses a distinct study-specific combined morphological outcome in second permanent molars among adolescents. The related manuscripts and their current submission status will be disclosed to the journal.

## 3. Results

### 3.1. Participant Flow and Characteristics

**Figure 1:**
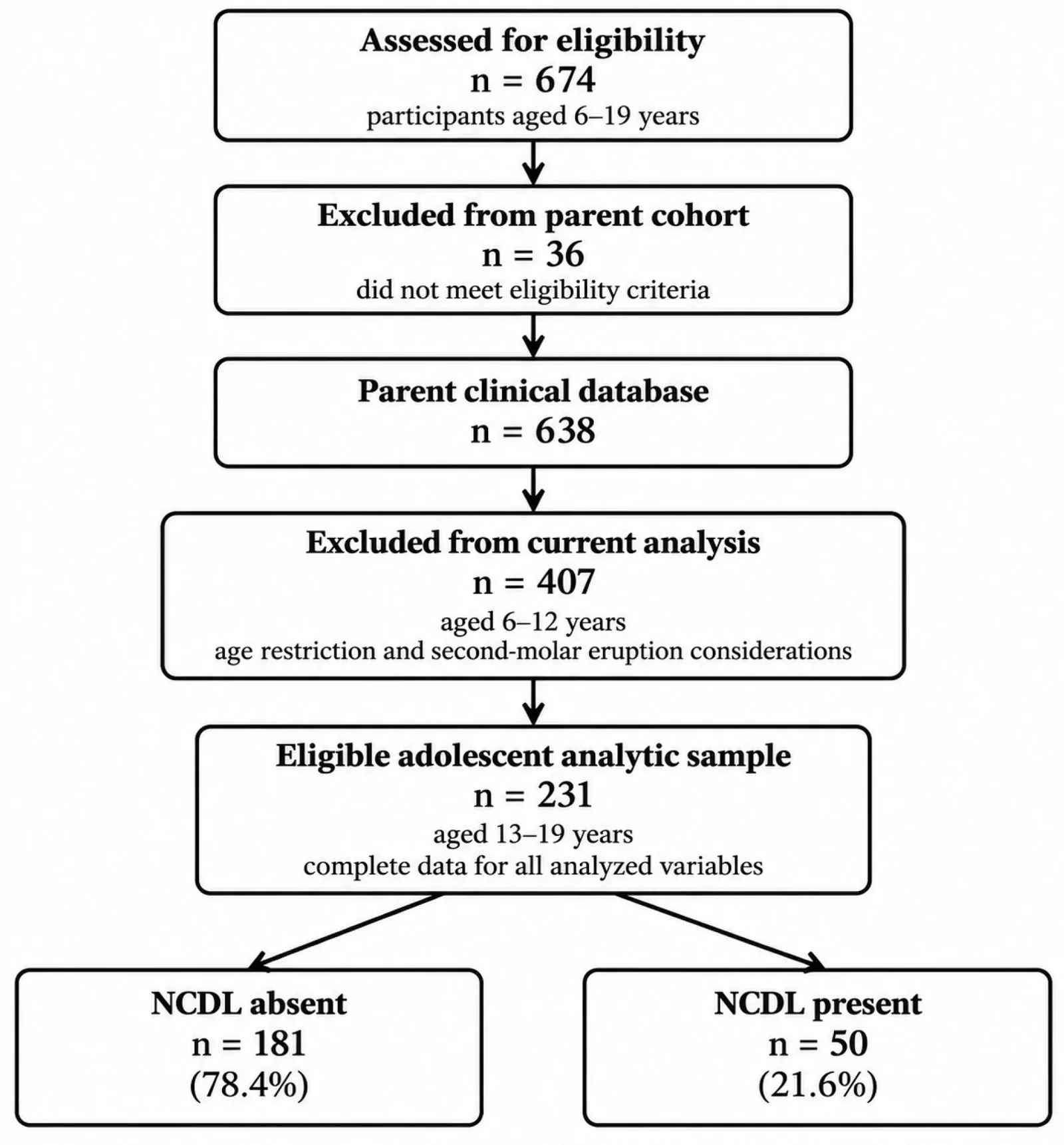
Participant flow and construction of the analytic sample.

Between April 2025 and April 2026, 674 children and adolescents were assessed for eligibility across the four participating dental care settings. Thirty-six individuals were excluded because they did not meet one or more predefined eligibility criteria, leaving 638 participants in the parent clinical database. After restricting the current post hoc secondary analysis to adolescents, 407 participants aged 6–12 years were excluded, and 231 adolescents aged 13–19 years comprised the analytic sample (Figure 1). There were no missing data for the analyzed variables. The demographic and clinical characteristics of the analytic sample are summarized in Table 1. The mean age was 14.64 years (SD 1.52), 119 participants (51.5%) were girls, 103 (44.6%) lived in rural areas, and 82 participants were recruited from each of Dolj and Olt counties, with 67 from Gorj. Exact participant counts for the two Dolj recruitment centers cannot be reconstructed from the final analytical dataset because center identity was not retained separately from county.

### 3.2. Frequency of the Study-Specific Morphological Outcome and Unadjusted Clinical Comparisons

This was an internal analytical coding-consistency check only. It did not involve comparison of the analytical variables with the original examination forms and therefore should not be interpreted as a raw-source audit; it does not establish diagnostic validity or outcome-specific reproducibility. Unadjusted comparisons of oral clinical indicators between participants with and without the study-specific combined morphological outcome are presented in Table 2.

### 3.3. Associations with Sex, Residence, and County

The frequency of the study-specific combined morphological outcome differed significantly by sex. The outcome was present in 34/112 boys (30.4%) and 16/119 girls (13.4%), corresponding to a small-to-moderate unadjusted association (chi-squared (1) = 9.73, *p* = 0.002, phi = 0.205). The unadjusted odds of the study-specific morphological outcome were approximately 2.81 times higher in boys than in girls (95% CI 1.45–5.45). The frequency of the outcome did not differ significantly by residential environment or county (Table 3).

### 3.4. Correlation Structure

The study-specific morphological outcome showed weak positive correlations with DMF-T (Spearman rho = 0.133, *p* = 0.044) and CPI (rho = 0.157, *p* = 0.017), but not with PI, GI, or age. The oral clinical indices were strongly intercorrelated, supporting a parsimonious primary model and cautioning against post hoc mechanistic interpretation.

### 3.5. Primary Multivariable Logistic Regression

**Figure 2:**
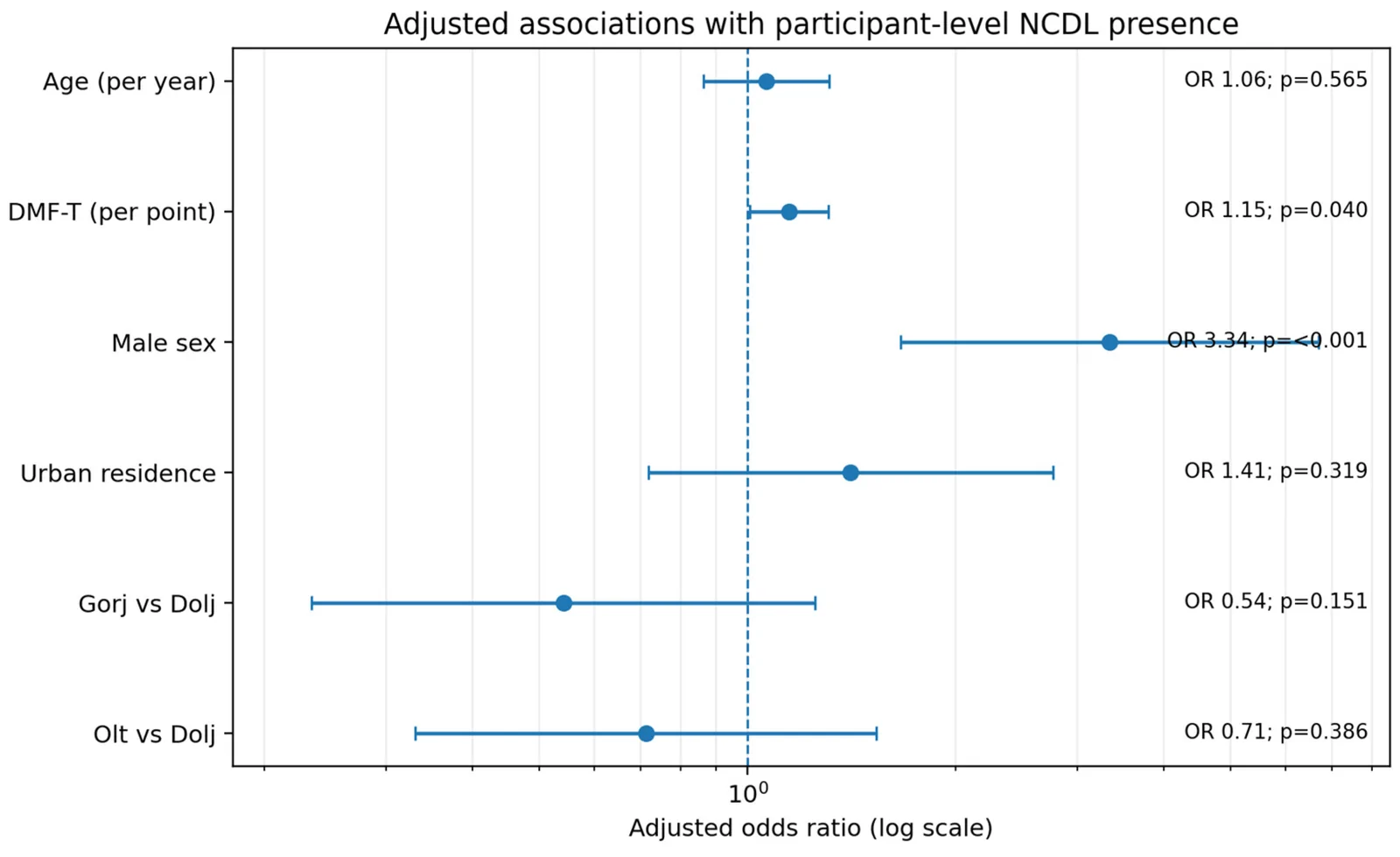
Adjusted odds ratios and 95% confidence intervals from the binary logistic regression model for the participant-level study-specific combined morphological outcome. The vertical reference line indicates an odds ratio of 1.00. Reference categories were female sex, rural residence, and Dolj County.

The primary logistic regression model including DMF-T, age, sex, residence, and county was significant compared with the intercept-only model (likelihood-ratio chi-squared (6) = 18.15, *p* = 0.006; Nagelkerke R2 = 0.117). Male sex (OR = 3.342, 95% CI 1.665–6.707, *p* < 0.001) and DMF-T (OR = 1.148 per point, 95% CI 1.006–1.309, *p* = 0.040) were associated with the study-specific morphological outcome after adjustment; age, residence, and county were not (Figure 2, Table 4).

The primary model had modest discrimination (AUC = 0.700) and very low sensitivity at the default 0.50 threshold; these metrics are reported descriptively and do not support clinical prediction or screening use.

### 3.6. Sensitivity Analysis Excluding Local DMF-T Overlap

After removing the D/M/F contribution of teeth 17, 27, 37, and 47 from total DMF-T, the adjusted association remained positive but was borderline: DMFT_excl_2M OR = 1.198 per point (95% CI 1.000–1.435, *p* = 0.049). Male sex remained associated with the outcome (OR = 3.310, 95% CI 1.647–6.652, *p* < 0.001), whereas age, residence, and county remained non-significant. The sensitivity model was significant overall (likelihood-ratio chi-squared (6) = 17.538, *p* = 0.007; Nagelkerke R2 = 0.113), had AUC = 0.695, and showed no multicollinearity (VIF 1.025–1.067). The similarity of the point estimates suggests that the primary association was not solely generated by direct overlap of the four evaluated molars with DMF-T, although the confidence interval touching 1.000 reinforces the exploratory and borderline nature of the finding (Supplementary Table S2).

### 3.7. Secondary DMF-T Models

In the secondary Poisson model treating DMF-T as the outcome, the study-specific morphological outcome was associated with an estimated 18.3% higher DMF-T rate after adjustment for age, sex, residence, and county (IRR = 1.183, 95% CI 1.027–1.359, *p* = 0.019). However, the deviance and Pearson goodness-of-fit tests were significant, and the dispersion ratios were above 1 (deviance/df = 1.59; Pearson/df = 1.34), indicating inadequate Poisson fit. Because of inadequate model fit, the Poisson estimate was not used for inferential interpretation.

The Gaussian identity-link sensitivity model estimated an adjusted mean DMF-T difference of 0.86 points for adolescents with versus without the study-specific morphological outcome (95% CI 0.03–1.69, *p* = 0.043). Residual plots showed no severe heteroscedasticity or observations with standardized residuals beyond ±3, and VIFs were close to 1. Nevertheless, the global Gaussian model did not improve AIC or BIC relative to the null model and was not statistically significant as a whole. The sensitivity analysis was interpreted cautiously (Table 5).

## 4. Discussion

### 4.1. Principal Findings

This exploratory cross-sectional secondary analysis found a study-specific combined morphological change on at least one second permanent molar in 50 of 231 adolescents. Male sex and greater cumulative caries experience were associated with the outcome in the primary adjusted model. The sensitivity model excluding the D/M/F contribution of teeth 17, 27, 37, and 47 produced a similar but borderline DMF-T estimate (OR = 1.198, 95% CI 1.000–1.435), indicating that local tooth overlap alone does not explain the observed direction of association. The effect remains modest and should be interpreted as hypothesis-generating rather than confirmatory.

The findings do not establish a shared biological pathway between caries and non-carious tooth surface loss [26,27]. Recent adolescent studies using validated BEWE scoring demonstrate associations of erosive tooth wear with age, sex, caries status, and behavioral exposures, but these studies also underscore the importance of standardized severity and surface recording [14,15]. Caries is itself a multifactorial disease with a substantial global burden and complex ecological and biological determinants [28,29,30,31,32,33,34,35,36,37]. Because diet, acidic beverage exposure, reflux, fluoride exposure, brushing technique, bruxism, socioeconomic status, and dental care use were not measured here, the present study cannot identify mechanisms or support an integrated oral risk profile explanation.

The association with male sex persisted after adjustment and in the sensitivity model. This should be interpreted as a dataset-specific association rather than evidence of a direct sex effect, because relevant behavioral, dietary, functional, and socioeconomic variables were not measured. The contemporary non-carious cervical lesion and tooth wear literature emphasizes heterogeneous morphology, multifactorial mechanisms, and exposure-related differences [38,39,40,41,42,43,44,45,46,47]. Causal attribution therefore requires direct measurement of these factors [14,26].

The study-specific outcome was also associated with higher CPI in the unadjusted analysis, whereas the PI and GI differences were not statistically significant. Because CPI, PI, GI, and DMF-T were strongly intercorrelated, and only 50 outcome events were available, these oral indices were not entered together into the primary model. These secondary comparisons are exploratory and do not establish a plaque-, gingival-, or periodontal-mediated pathway.

Direct comparison of the observed 21.6% with published tooth wear prevalence is inappropriate. The present endpoint is restricted to four second permanent molars and combines several morphology-based patterns without a validated severity index. Age-related eruption and maturation of permanent teeth may further influence tooth-level comparisons in adolescents [48,49]. Recent systematic and adolescent evidence illustrates substantial variation in morphology, diagnostic thresholds, indices, and examined surfaces [12,14,20,26]. Accordingly, 21.6% is reported only as the frequency of this study-specific endpoint within the clinical sample, not as a standardized NCDL or whole-mouth tooth wear prevalence estimate.

County and residence were not associated with the outcome in the present models, but this does not establish equivalence between locations. Recruitment occurred in four clinical settings, including two centers in Dolj County, while the analytical dataset retained county but not participant-level center. Center-specific recruitment mix and care-seeking therefore remain uncontrolled sources of heterogeneity. More broadly, recent European caries evidence emphasizes the influence of geographical and socioeconomic context on observed caries experience [3,27].

The logistic model was used for adjusted association estimation only. Established methodological guidance supports cautious interpretation of agreement and multivariable model performance [50,51,52,53,54,55]. Its modest AUC and very low sensitivity at the default threshold reinforce that the analysis should not be interpreted as a screening or prediction model.

Secondary reciprocal models treating DMF-T as the outcome were retained only as supportive sensitivity analyses because the Poisson model showed inadequate fit and the Gaussian model was weak globally. They are not used to strengthen causal or predictive interpretation.

### 4.2. Strengths and Limitations

Strengths include an age-restricted permanent-dentition sample, explicit participant-level construction from tooth-level study-specific morphological records, complete data, standardized data collection by a single trained examiner, reporting of effect sizes and confidence intervals, and transparent evaluation of both association and model performance.

Several major limitations should be considered. First, the cross-sectional, post hoc secondary design precludes temporal or causal inference. Second, consecutive non-probability recruitment from dental care settings limits representativeness. Third, the primary endpoint was a study-specific combined morphological outcome restricted to four second permanent molars, not a validated whole-mouth tooth wear diagnosis; BEWE or the Smith and Knight index was not applied, and no validated minimum severity/depth threshold was recorded. Subtype, surface, and severity were not retained separately. Fourth, outcome-specific reproducibility could not be established: only a historical global kappa of 0.86 was retained, whereas the paired repeated records needed to calculate NCDL-specific agreement, kappa with 95% CI, or index-specific ICCs are unavailable. This is a major limitation of the primary endpoint. Fifth, recruitment center was not retained separately from county, preventing center-stratified description or center fixed-effect sensitivity analysis; adjustment for county does not fully address the two distinct Dolj centers. Sixth, the same examiner recorded the study-specific morphology and the caries/periodontal indicators and was not blinded to the other findings, creating potential observer-related bias. Seventh, age 13–19 years spans meaningful differences in post-eruptive exposure time; age was adjusted for, but residual age-related heterogeneity may remain. Eighth, important exposures including diet, acidic beverages, reflux, fluoride, brushing technique and force, bruxism, socioeconomic status, and dental care use were unavailable. Finally, only 50 participants were outcome-positive, the primary DMF-T association was borderline, and secondary analyses were exploratory without multiplicity adjustment. Previous literature also reports sex-related differences in periodontal and gingival conditions in young people [56,57]. Preventive oral-health evidence identifies screening, common risk factors, diet, fluoride exposure, and sugar-related behaviors as important contextual determinants in children and adolescents [58,59,60,61,62,63,64,65].

Participants were recruited from four dental care settings, and potential center-level differences or clustering were not modeled. Adjustment for county may not fully account for differences between the two participating centers in Dolj County.

### 4.3. Generalizability

The results apply most directly to adolescents attending the participating dental care settings in Southwest Romania. They should not be interpreted as a national Romanian prevalence estimate or generalized to younger children, adults, or populations examined with validated tooth wear indices. Replication using probability-based sampling and standardized whole-mouth measures is required.

## 5. Conclusions

A modest association was observed between cumulative caries experience and a study-specific combined morphological outcome on second permanent molars in a minimally adjusted exploratory analysis. The association remained positive but borderline after excluding the same four molars from DMF-T. Given the non-standardized outcome, unavailable outcome-specific reliability, clinical sampling frame, unmodeled center effects, and unmeasured behavioral and dietary exposures, the finding requires confirmation using validated tooth wear measures and population-based longitudinal data. This study does not support causal, prevalence-generalization, screening, or predictive claims.

## Figures and Tables

**Table 1:** Participant characteristics and distribution of the study-specific combined morphological outcome.

Characteristic	Value
Analytic sample	231
Age, years	14.64 ± 1.52; median 14; range 13–19
Female sex	119 (51.5%)
Male sex	112 (48.5%)
Rural residence	103 (44.6%)
Urban residence	128 (55.4%)
County: Dolj/Gorj/Olt	82 (35.5%)/67 (29.0%)/82 (35.5%)
DMF-T	4.93 ± 2.58; median 5; IQR 4; range 0–10
Caries present (DMF-T > 0)	223 (96.5%)
PI	1.835 ± 0.847
GI	1.382 ± 0.728
CPI	1.030 ± 0.831
Any study-specific morphological change on 17/27/37/47	50 (21.6%)
Morphological change tooth 17	14 (6.1%)
Morphological change tooth 27	15 (6.5%)
Morphological change tooth 37	12 (5.2%)
Morphological change tooth 47	9 (3.9%)

Note. Values are *n* (%) or mean ± SD unless otherwise specified. DMF-T: Decayed, Missing, and Filled Teeth; PI: Plaque Index; GI: Gingival Index; CPI: Community Periodontal Index.

**Table 2:** Oral clinical indicators according to the study-specific combined morphological outcome.

Variable	Morphological Outcome Absent *n* = 181	Morphological Outcome Present *n* = 50	Mann–Whitney U	*p*	Rank-Biserial Correlation (95% CI)
DMF-T	4.76 ± 2.59; median 5	5.54 ± 2.48; median 6	3689	0.044	0.185 (0.006 to 0.352)
CPI	0.96 ± 0.85	1.28 ± 0.73	3587	0.017	0.207 (0.029 to 0.373)
PI	1.79 ± 0.86	2.00 ± 0.79	3930	0.155	0.132 (−0.049 to 0.304)
GI	1.35 ± 0.74	1.51 ± 0.69	3954	0.173	0.126 (−0.054 to 0.299)
Age, years	14.61 ± 1.52; median 14	14.76 ± 1.55; median 14	4238	0.481	0.063 (−0.117 to 0.240)

Note. Unadjusted two-sided comparisons.

**Table 3:** Study-specific combined morphological outcome across demographic strata.

Factor	Category	Morphological Outcome Present, *n*/*N* (%)	chi-Squared (df)	*p*	Effect Size
Sex	Girls	16/119 (13.4%)	9.730 (1)	0.002	Phi = 0.205
	Boys	34/112 (30.4%)			
Residence	Rural	20/103 (19.4%)	0.544 (1)	0.461	Phi = 0.049
	Urban	30/128 (23.4%)			
County	Dolj	21/82 (25.6%)	1.351 (2)	0.509	Cramer’s V = 0.076
	Gorj	12/67 (17.9%)			
	Olt	17/82 (20.7%)			

Note. Percentages are calculated within each demographic category. Expected counts were adequate for Pearson chi-squared testing.

**Table 4:** Adjusted logistic regression for the study-specific combined morphological outcome.

Predictor	Adjusted OR	95% CI	*p*
Age, per year	1.064	0.862–1.313	0.565
DMF-T, per point	1.148	1.006–1.309	0.040
Male vs. female	3.342	1.665–6.707	<0.001
Urban vs. rural	1.409	0.718–2.764	0.319
Gorj vs. Dolj	0.541	0.234–1.252	0.151
Olt vs. Dolj	0.712	0.330–1.535	0.386

Note. Outcome coded 1 when the study-specific combined morphological outcome was present. Model likelihood-ratio chi-squared (6) = 18.147, *p* = 0.006; Nagelkerke R2 = 0.117. Reference categories: female sex, rural residence, and Dolj County.

**Table 5:** Secondary models evaluating DMF-T as the outcome.

Model	Outcome Scale	Morphological-Outcome Estimate	95% CI	*p*	Interpretation
Poisson log-link	DMF-T count	IRR 1.183	1.027–1.359	0.019	Inadequate global fit; supportive only
Gaussian identity-link	DMF-T continuous	Adjusted difference 0.860	0.034–1.686	0.043	Residual diagnostics acceptable; global model weak

Note. Both models were adjusted for age, sex, residence, and county. The primary inferential model for the manuscript was the logistic regression, with the study-specific combined morphological outcome as the dependent variable.

## Data Availability

The de-identified data supporting the findings are available from the corresponding authors on reasonable request, subject to ethical and institutional requirements.

## Supplementary Materials

The following supporting information can be downloaded at: https://www.mdpi.com/article/10.3390/jcm15176931/s1, Table S1: Participant-level analytical coding-consistency check for the study-specific combined morphological outcome in second permanent molars; Table S2: Sensitivity logistic regression analysis using DMF-T recalculated after excluding the D/M/F contribution of teeth 17, 27, 37, and 47.

## References

[1] World Health Organization (2022). Global Oral Health Status Report: Towards Universal Health Coverage for Oral Health by 2030.

[2] Wen P.Y.F., Chen M.X., Zhong Y.J., Dong Q.Q., Wong H.M. (2022). Global burden and inequality of dental caries, 1990 to 2019. J. Dent. Res..

[3] Skeie M.S., Sen A., Dahllof G., Fagerhaug T.N., Hovik H., Klock K.S. (2022). Dental caries at enamel and dentine level among European adolescents—A systematic review and meta-analysis. BMC Oral Health.

[4] World Health Organization (2013). Oral Health Surveys: Basic Methods.

[5] Broadbent J.M., Thomson W.M. (2005). For debate: Problems with the DMF index pertinent to dental caries data analysis. Community Dent. Oral Epidemiol..

[6] Ismail A.I., Sohn W., Tellez M., Amaya A., Sen A., Hasson H., Pitts N.B. (2007). The International Caries Detection and Assessment System (ICDAS): An integrated system for measuring dental caries. Community Dent. Oral Epidemiol..

[7] Nyvad B., Machiulskiene V., Baelum V. (1999). Reliability of a new caries diagnostic system differentiating between active and inactive caries lesions. Caries Res..

[8] Levitch L.C., Bader J.D., Shugars D.A., Heymann H.O. (1994). Non-carious cervical lesions. J. Dent..

[9] Bartlett D.W., Shah P. (2006). A critical review of non-carious cervical lesions and the role of abfraction, erosion, and abrasion. J. Dent. Res..

[10] Wood I., Jawad Z., Paisley C., Brunton P. (2008). Non-carious cervical tooth surface loss: A literature review. J. Dent..

[11] Grippo J.O., Simring M., Coleman T.A. (2012). Abfraction, abrasion, biocorrosion, and the enigma of noncarious cervical lesions: A 20-year perspective. J. Esthet. Restor. Dent..

[12] Garay Villamayor K.G., Codas-Duarte D., Ramirez I., Souza-Gabriel A.E., Sousa-Neto M.D., Candemil A.P. (2024). Morphological characteristics of non-carious cervical lesions. A systematic review. Arch. Oral Biol..

[13] Kitasako Y., Ikeda M., Takagaki T., Burrow M.F., Tagami J. (2021). The prevalence of non-carious cervical lesions with or without erosive etiological factors among adults of different ages in Tokyo. Clin. Oral Investig..

[14] Avila V., Beltran E.O., Cortes A., Usuga-Vacca M., Parras J.E.C., Diaz-Baez D., Martignon S. (2024). Prevalence of erosive tooth wear and associated risk factors in Colombian adolescents. Braz. Oral Res..

[15] Rusyan E., Grabowska E., Struzycka I. (2022). The association between erosive tooth wear and diet, hygiene habits and health awareness in adolescents aged 15 in Poland. Eur. Arch. Paediatr. Dent..

[16] von Elm E., Altman D.G., Egger M., Pocock S.J., Gøtzsche P.C., Vandenbroucke J.P., Initiative S. (2007). The Strengthening the Reporting of Observational Studies in Epidemiology (STROBE) statement: Guidelines for reporting observational studies. Lancet.

[17] Vandenbroucke J.P., von Elm E., Altman D.G., Gøtzsche P.C., Mulrow C.D., Pocock S.J., Poole C., Schlesselman J.J., Egger M., STROBE initiative (2007). Strengthening the Reporting of Observational Studies in Epidemiology (STROBE): Explanation and elaboration. Ann. Intern. Med..

[18] Bartlett D., Ganss C., Lussi A. (2008). Basic Erosive Wear Examination (BEWE): A new scoring system for scientific and clinical needs. Clin. Oral Investig..

[19] Smith B.G.N., Knight J.K. (1984). An index for measuring the wear of teeth. Br. Dent. J..

[20] Pineda A.E.G.-A., Garcia-Perez A. (2023). Incidence and progression of erosive tooth wear according to tooth type in schoolchildren of Mexico City. J. Clin. Pediatr. Dent..

[21] Ainamo J., Barmes D., Beagrie G., Cutress T., Martin J., Sardo-Infirri J. (1982). Development of the World Health Organization Community Periodontal Index of Treatment Needs. Int. Dent. J..

[22] Silness J., Loe H. (1964). Periodontal disease in pregnancy II. Correlation between oral hygiene and periodontal condition. Acta Odontol. Scand..

[23] Loe H., Silness J. (1963). Periodontal disease in pregnancy I. Prevalence and severity. Acta Odontol. Scand..

[24] Preisser J.S., Stamm J.W., Long D.L., Kincade M.E. (2012). Review and recommendations for zero-inflated count regression modeling of dental caries indices in epidemiological studies. Caries Res..

[25] Preisser J.S., Long D.L., Stamm J.W. (2017). Matching the statistical model to the research question for dental caries indices with many zero counts. Caries Res..

[26] Dallavilla G.G., Martins D.S., Peralta-Mamani M., Santiago Junior J.F., Rios D., Honorio H.M. (2024). Prevalence of erosive tooth wear in risk group patients: Systematic review. Clin. Oral Investig..

[27] Veenman F., van Dijk A., Arredondo A., Medina-Gomez C., Wolvius E., Rivadeneira F., Àlvarez G., Blanc V., Kragt L. (2024). Oral microbiota of adolescents with dental caries: A systematic review. Arch. Oral Biol..

[28] Peres M.A., Macpherson L.M.D., Weyant R.J., Daly B., Venturelli R., Mathur M.R., Listl S., Celeste R.K., Guarnizo-Herreño C.C., Kearns C. (2019). Oral diseases: A global public health challenge. Lancet.

[29] Bernabe E., Marcenes W., Hernandez C.R., Bailey J., Abreu L.G., Alipour V., Amini S., Arabloo J., Arefi Z., GBD 2017 Oral Disorders Collaborators (2020). Global, regional, and national levels and trends in burden of oral conditions from 1990 to 2017: A systematic analysis for the Global Burden of Disease 2017 study. J. Dent. Res..

[30] Frencken J.E., Sharma P., Stenhouse L., Green D., Laverty D., Dietrich T. (2017). Global epidemiology of dental caries and severe periodontitis: A comprehensive review. J. Clin. Periodontol..

[31] Pitts N.B., Zero D.T., Marsh P.D., Ekstrand K., Weintraub J.A., Ramos-Gomez F., Tagami J., Twetman S., Tsakos G., Ismail A. (2017). Dental caries. Nat. Rev. Dis. Primers.

[32] Selwitz R.H., Ismail A.I., Pitts N.B. (2007). Dental caries. Lancet.

[33] Fejerskov O. (2004). Changing paradigms in concepts on dental caries: Consequences for oral health care. Caries Res..

[34] Marsh P.D. (2006). Dental plaque as a biofilm and a microbial community: Implications for health and disease. BMC Oral Health.

[35] Takahashi N., Nyvad B. (2011). The role of bacteria in the caries process: Ecological perspectives. J. Dent. Res..

[36] Featherstone J.D.B. (2004). The continuum of dental caries: Evidence for a dynamic disease process. J. Dent. Res..

[37] Zero D.T., Fontana M., Martínez-Mier E.A., Ferreira-Zandoná A., Ando M., González-Cabezas C., Bayne S. (2009). The biology, prevention, diagnosis and treatment of dental caries: Scientific advances in the United States. J. Am. Dent. Assoc..

[38] Penoni D.C., Miranda M.E.S.N.G., Sader F., Vettore M.V., Leao A.T.T. (2021). Factors associated with noncarious cervical lesions in different age ranges: A cross-sectional study. Eur. J. Dent..

[39] Aw T.C., Lepe X., Johnson G.H., Mancl L. (2002). Characteristics of noncarious cervical lesions: A clinical investigation. J. Am. Dent. Assoc..

[40] Osborne-Smith K.L., Burke F.J.T., Wilson N.H.F. (1999). The aetiology of the non-carious cervical lesion. Int. Dent. J..

[41] Sarode G.S., Sarode S.C. (2013). Abfraction: A review. J. Oral Maxillofac. Pathol..

[42] Rees J.S. (2006). The biomechanics of abfraction. Proc. Inst. Mech. Eng. H.

[43] Shellis R.P., Addy M. (2014). The interactions between attrition, abrasion and erosion in tooth wear. Monographs in Oral Science.

[44] Lussi A., Carvalho T.S. (2014). Erosive tooth wear: A multifactorial condition of growing concern and increasing knowledge. Monographs in Oral Science.

[45] Schlueter N., Luka B. (2018). Erosive tooth wear: A review on global prevalence and on its prevalence in risk groups. Br. Dent. J..

[46] Racki D.N.O., Comim L.D., Dalla Nora A., Zenkner J.E.A., Alves L.S. (2021). Is Toothbrush Bristle Stiffness Associated with Erosive Tooth Wear in Adolescents? Findings from a Population-Based Cross-Sectional Study. Caries Res..

[47] Jaeggi T., Lussi A. (2014). Prevalence, incidence and distribution of erosion. Monographs in Oral Science.

[48] Almonaitiene R., Balciuniene I., Tutkuviene J. (2010). Factors influencing permanent teeth eruption. Part one: General factors. Stomatologija.

[49] Moorrees C.F.A., Fanning E.A., Hunt E.E. (1963). Age variation of formation stages for ten permanent teeth. J. Dent. Res..

[50] Cohen J. (1960). A coefficient of agreement for nominal scales. Educ. Psychol. Meas..

[51] Landis J.R., Koch G.G. (1977). The measurement of observer agreement for categorical data. Biometrics.

[52] Hosmer D.W., Lemeshow S., Sturdivant R.X. (2013). Applied Logistic Regression.

[53] Steyerberg E.W. (2019). Clinical Prediction Models: A Practical Approach to Development, Validation, and Updating.

[54] Saito T., Rehmsmeier M. (2015). The precision-recall plot is more informative than the ROC plot when evaluating binary classifiers on imbalanced datasets. PLoS ONE.

[55] Van Calster B., McLernon D.J., van Smeden M., Wynants L., Steyerberg E.W. (2019). Calibration: The Achilles heel of predictive analytics. BMC Med..

[56] Shiau H.J., Reynolds M.A. (2010). Sex differences in destructive periodontal disease: A systematic review. J. Periodontol..

[57] Furuta M., Ekuni D., Irie K., Azuma T., Tomofuji T., Ogura T., Morita M. (2011). Sex differences in gingivitis relate to interaction of oral health behaviors in young people. J. Periodontol..

[58] Chou R., Bougatsos C., Griffin J., Selph S.S., Ahmed A., Fu R., Nix C., Schwarz E. (2023). Screening, Referral, Behavioral Counseling, and Preventive Interventions for Oral Health in Children and Adolescents Aged 5 to 17 Years: A Systematic Review for the US Preventive Services Task Force. JAMA.

[59] Twetman S. (2018). Prevention of dental caries as a non-communicable disease. Eur. J. Oral Sci..

[60] Sheiham A., Watt R.G. (2000). The common risk factor approach: A rational basis for promoting oral health. Community Dent. Oral Epidemiol..

[61] Watt R.G., Daly B., Allison P., Macpherson L.M.D., Venturelli R., Listl S., Weyant R.J., Mathur M.R., Guarnizo-Herreño C.C., Celeste R.K. (2019). Ending the neglect of global oral health: Time for radical action. Lancet.

[62] Cascaes A.M., da Silva N.R.J., Fernandez M.D.S., Bomfim R.A., Vaz J.D.S. (2023). Ultra-processed food consumption and dental caries in children and adolescents: A systematic review and meta-analysis. Br. J. Nutr..

[63] Walsh T., Worthington H.V., Glenny A.M., Marinho V.C.C., Jeroncic A. (2019). Fluoride toothpastes of different concentrations for preventing dental caries. Cochrane Database Syst. Rev..

[64] Moynihan P., Petersen P.E. (2004). Diet, nutrition and the prevention of dental diseases. Public Health Nutr..

[65] Luo B.W., Liang N.L., Townsend J.A., Lo E.C.M., Chu C.H., Duangthip D. (2024). Sugar substitutes on caries prevention in permanent teeth among children and adolescents: A systematic review and meta-analysis. J. Dent..

