# Supplementary material for: Study-Specific Morphological Signs of Non-Carious Tooth Surface Loss in Second Permanent Molars and Caries Experience in a Clinical Sample of Adolescents from Southwest Romania: A Multicenter Cross-Sectional Study
Source: J Clin Med. 2026 Sep 7;15(17):6931. doi: 10.3390/jcm15176931 (PMC13566170; doi:10.3390/jcm15176931)
Supplement: Supplementary file 1 [file jcm-15-06931-s001.zip › jcm-4485923-supplementary.pdf]

Supplementary Material

Study-Specific Morphological Signs of Non-Carious Tooth Surface Loss in Second Permanent Molars and Caries Experience in a Clinical Sample of Adolescents from Southwest Romania: A Multicenter Cross-Sectional Study

Supplementary Table S1. Participant-level analytical coding-consistency check for the study-specific combined morphological outcome in second permanent molars.

Variable	Category / tooth	n	% of n=231
NCDL_count	0 positive evaluated molars	181	78.4
NCDL_count	1 positive evaluated molar	50	21.6
NCDL_count	2-4 positive evaluated molars	0	0.0
Tooth-specific binary variable	Tooth 17 positive	14	6.1
Tooth-specific binary variable	Tooth 27 positive	15	6.5
Tooth-specific binary variable	Tooth 37 positive	12	5.2
Tooth-specific binary variable	Tooth 47 positive	9	3.9

Note. The tooth-specific positive counts sum to 50 (14+15+12+9), exactly matching the 50 participant-level positive outcomes. The audit confirms internal consistency of the analytical coding; it does not establish diagnostic validity or outcome-specific reproducibility.

Supplementary Table S2. Sensitivity logistic regression analysis using DMF-T recalculated after excluding the D/M/F contribution of teeth 17, 27, 37, and 47.

Predictor	Adjusted OR	95% CI	p
DMFT_excl_2M, per point	1.198	1.000-1.435	0.049
Age, per year	1.065	0.872-1.301	0.536
Male vs. female	3.310	1.647-6.652	<0.001
Urban vs. rural	1.428	0.738-2.765	0.291
Gorj vs. Dolj	0.543	0.240-1.225	0.141
Olt vs. Dolj	0.727	0.337-1.566	0.415

Model statistics: likelihood-ratio chi-squared (6)=17.538, p=0.007; McFadden R2=0.073; Nagelkerke R2=0.113; Tjur R2=0.077; AUC=0.695; accuracy=0.771; sensitivity=0.020; specificity=0.978. Multicollinearity was low (VIF range 1.025-1.067).

Note. DMFT_excl_2M was calculated by removing the D/M/F contribution of teeth 17, 27, 37, and 47 from total permanent-dentition DMF-T. The model otherwise used the same covariates and reference categories as the primary logistic regression.
